# Oncology nurses’ perceptions of work stress and its sources in a university‐teaching hospital: A qualitative study

**DOI:** 10.1002/nop2.192

**Published:** 2018-08-16

**Authors:** Dhuha Youssef Wazqar

**Affiliations:** ^1^ Head of Medical Surgical Nursing Department, Acting Head of Critical Care and Emergency Nursing, Faculty of Nursing King Abdulaziz University Jeddah Saudi Arabia

**Keywords:** content analysis, cultural difference, language barriers, oncology nurses, people with cancer, qualitative study, Saudi Arabia, work stress, workload

## Abstract

**Aim:**

To explore and understand work stress and its sources among oncology nurses in a Saudi university‐teaching hospital.

**Design:**

Qualitative descriptive study using semistructured interviews.

**Methods:**

Fourteen oncology nurses working in a university‐teaching hospital were interviewed between October ‐ December 2016. Qualitative content analysis according to the Krippendorff method was used to explore work‐related stressors among oncology nurses in Saudi Arabia.

**Results:**

Two categories were emerged including “extent of work stress” and “work‐related stressors”. The second category included the following subcategories of workload and staff shortage, emotional demands, lack of social support, language barriers, and lack of respect from patients and family members and cultural differences.

## 1 INTRODUCTION

The topic of work stress in nursing has been documented for more than 40 years (Lazarus, 1966) and reported to be increasing in many countries, such as South Africa (Khamisa, Oldenburg, Peltzer, & Ilic, 2015), China, (Yau et al., 2012), India (Singh, 2013), and Middle East countries such as Saudi Arabia (SA) (Al‐Makhaita, Sabra, & Hafez, 2014; Wazqar, Kerr, Regan, & Orchard, 2017a). Work stress is the harmful physical and emotional reaction to a poor match between work overloads and the employee's abilities, resources, or needs (Lazarus & Folkman, 1984). Nursing is generally perceived as a stressful and demanding profession and nurses are more frequently exposed to stress‐provoking situations than other professions (Collins, 2000). Many studies on stress in nursing have attempted to examine the effects of work stress on nurses’ health and well‐being. There seems to be universal agreement that the experience of work stress commonly has an impact on the quality of nurses’ working lives, increases minor psychiatric illness and may lead to some forms of physical disease and behavioural problems (Mckinney, 2011; Mojoyinola, 2008). Work stress affects not only the health and well‐being of the individual it can also have adverse consequences for patients and healthcare organization for which the individual works, through reduced productivity, absenteeism, and turnover. Researchers found that work stress is associated with lower morale, reduced work performance, higher job turnover, decreased job satisfaction, loss of productivity, high rates of absenteeism, and reduced quality of nursing care for patients (Mckinney, 2011; Mojoyinola, 2008; Nabirye, Brown, Pryor, & Maples, 2011; Wazqar et al., 2017a). Some of quantitative studies have paid attention to examine the work stress in oncology nurses in different countries. In these studies, several work‐related stressors specific to oncology nurses have been identified, such as administering aggressive cancer treatments, intensive involvement with highly demanding patients and families, dealing with the death of patients, developing close relationships with patients during long‐term hospitalizations, communication issues, poor relationships with medical staff, ethical and moral issues related to patient care and research, interpersonal staff conflicts, workload, finding a balance between one's individual and professional life, and lack of in‐service training (Bardeh, Naji, & Zarea, 2016; Ko & Kiser‐Larson, 2016; Zareifar et al., 2017). In a study among Iranian oncology nurses, researchers indicated that role conflicts, lack of control and organizational barriers, such as oncology nurse shortages, facility and equipment limitations, and underpayment were significant sources of work stress which negatively affected the quality of nursing care (Iranmanesh, Raseveralyyani, & Forouzy, 2012). Also, a lack of support and understanding from senior staff was identified as one of the most stressful aspects among oncology nurses (Zander, Hutton, & King, 2013). According to another study conducted by Brajtman, Fothergill‐Bourbonnais, Casey, Alain, and Fiset (2007), researchers stated that feeling inadequately prepared to meet the emotional demands of patients and their families, lack of experience and lack of confidence providing appropriate care to dying patients were the greatest sources of work stress for oncology nurses. This burden may become overwhelming during situations where oncology nurses lack experience, skills, control, and social support to manage their own psychological health (Sabo, 2008). This study seeks to explore work‐related stressors faced by oncology nurses in a Saudi university‐teaching hospital and to understand oncology nursing stress using Lazarus and Folkman's (1984) transactional stress theory.

## 2 BACKGROUND

The transactional stress theory developed by Lazarus and Folkman (1984) was used as the theoretical framework for this study. The interaction between individual and environment, which working conditions create a feeling of being stressed, is the foundation of Lazarus’ cognitive theory of stress. An individual, according to this theory, has a cognitive assessment of threats that come from the environment. This cognitive assessment is called appraisal. The degree to which an individual appraises stress as a serious threat reveals the degree of stress experienced. However, “stress is not a property of the person, or of the environment, but arises when there is conjunction between a particular kind of environment and a particular kind of person that leads to a threat appraisal” (Lazarus, 1991, p. 3). The term transaction indicates that neither the individual nor the environment is carrying stress. What carries either subjective or objective stress resides in the relationship between the environment and the individual. For example, work environment may include events called stimuli that are encountered by employees. The individuals' responses to these environmental stimuli are under the influence of their appraisal.

An individual in the encounter with a stressful condition make two appraisals: primary and secondary. The primary appraisal is a process by which individuals give meaning to an encounter and decide whether something significant to them is at stake or not. According to the transactional theory of stress (Lazarus & Folkman, 1984), if an individual evaluates the encounter as irrelevant, it will simply be ignored because it may have no personal significance for the individual. In addition, if people evaluate an encounter as benign, they will consider it as desirable or beneficial. Evaluating a situation or an event as a stressful encounter, on the other hand, indicates that the questioned event or situation is harmful, threatening, or challenging (Lazarus, 1994). The secondary appraisal begins when an encounter is appraised as a threat to the individual's well‐being. This process concerns the identification of coping resources to deal with the threat. Therefore, in this theory, the experience of stress depends not only on the quality of one's individual resources and the level of environmental threats but also on the quality of the interactions between individual and environment. The approach identifies the two processes as “critical mediators of stressful person‐environment relations and their immediate and long‐range outcomes” (Folkman, Lazarus, Dunkel‐Schetter, DeLongis, & Gruen, 1986, p. 992). Therefore, as Lazarus (1991) has discussed, stress is defined as the overall transaction process and not a particular variable in the individual or the environment. Additionally, by drawing on Lazarus and Folkman's (1984) theoretical framework, it was possible to conduct a study that described oncology nurses’ stress and it sources across their professional lives whereby a much fuller picture of work stress could be described in a Saudi healthcare context.

There were many different questionnaires developed for examining work stress in nursing. However, Polit and Beck (2012) have criticized the data collected through such instruments because of the low response rate and the superficial and artificial nature of such data. On the other hand, few investigators have paid attention to the life experiences of oncology nurses (in the field of work stress) and to the best of the researcher's knowledge, there is no qualitative study is published on work stress of oncology nurses in SA. Most studies examining work stress among oncology nurses were quantitative and conducted in Western and European countries. In addition, there are fundamental differences between the healthcare system and medical care services in SA with other developing and developed countries. Hence, conducting a qualitative study in this regard can help to clarify and extend understanding of work stress in oncology nursing. Considering the effects of work stress on oncology nurses' physical and mental health, lack of related studies and attention to individual perceptions, and experiences of oncology nurses in this area, this qualitative study explores the experiences and perceptions of oncology nurses in the field of work stress.

### 2.1 Aim

The aim of the study was to explore and understand work stress and its sources among oncology nurses working in a Saudi university‐teaching hospital. The following research questions guided the study: (a) To what extent do oncology nurses feel stress in their work? (b) What are the main work‐related stressors among oncology nurses working in a Saudi university‐teaching hospital?

## 3 THE STUDY

### 3.1 Design

This research builds on a previous quantitative study (Wazqar et al., 2017a) that employed Lazarus and Folkman's (1984) to examine job strain, coping, and work performance in oncology nurses working in Saudi hospitals. Wazqar et al. (2017b) found that oncology nurses working in SA experienced a considerable amount of job strain that has a negative impact on their coping skills and work performance. Consequently, a qualitative approach was adopted in a university‐teaching hospital in Jeddah, SA, to explore further why oncology nurses reported their workplace experiences in SA are stressful and to identify sources of work stress perceived by oncology nurses. Qualitative approach was chosen because it can generate rich descriptions of phenomena and help to investigate previously unexplored complex issues (Polit & Beck, 2012).

### 3.2 Sample/participants

A purposive sampling approach was used to select 14 participants. The study population included oncology Registered Nurses who have had a minimum of 1 year of experience in oncology nursing, work as full‐time staff for at least 6 months in the hospital, providing direct care to adult and paediatric people with cancer in outpatient clinics or inpatient oncology units. The more than 1 year experience criterion helped to ensure that participants were experienced enough to comment on oncology nursing practice and the work as full‐time staff for at least 6 months in the selected hospital criterion helped to ensure that participants be familiar with the healthcare setting, past the initial stress of working in a different or new setting. Oncology nurses working in nondirect care positions were excluded because of differences in stressors related to their largely nonclinical job responsibilities. Interviews have been conducted with one and/or two nurses from each oncology unit and clinic in the hospital. The total number of oncology nurses in the selected hospital was 40 nurses.

### 3.3 Data collection

As one of the key aims of qualitative research is to understand phenomena from the participants’ viewpoint (Wilkinson, Joffe, & Yardley, 2004), the semistructured interview was selected to collect data about 14 oncology nurses’ experiences of work stress. Data were collected between October ‐ December 2016. By visiting each oncology ward/clinic and explaining the research purpose, the oncology nurses met the inclusion criteria, were recognized and invited to participate in the study. Small group meetings with oncology nurses in each unit occurred to ensure they met the inclusion criteria and provide explanation and answers to any questions regarding the study before obtaining participants’ agreement to proceed. After receiving oncology nurses consent to participate in the study, the most appropriate time and location for the interviews were identified. A semistructured interview guide (Table 1) which consisted of four open‐ended questions was used to allow the participants expressing their detailed experiences and perceptions. For preparing the interview guide, at first a list of questions about the sources of work stress in oncology nursing were prepared based on the existing literature. Then, the interview guide was moderated by the guidance of three experts in oncology nursing and nursing education. Interviews started by asking the participants about their demographic information (gender, age, nationality, and years of experience, advanced preparation in oncology nursing and oncology unit they work in). Then, they were asked to express and explain their experiences and perceptions (work stress and related sources in oncology nursing) and asked further questions related to the answers provided by them. Duration of each interview varied from 25 to 35 min, because of their busy schedule and continued until no new codes were identified.

**Table 1:** Interview guide

To what extent do you feel stress in your work? What are the main stressors in your work setting? Please provide me an example of a work situation that really stressed you out? Can you please comment about your experience with Saudi people with cancer and their families, if you consider them as sources of work stress?

### 3.4 Ethical considerations

The researcher conducted the interviews after obtaining the University Ethical Review Board approval and participants’ consent. All participants received oral and written information about purpose, design, and advantages of the study. The interviews were audio recorded after receiving permission from the participant. If a participant did not provide permission, then only notes were taken. Participant were informed in advance that they can leave at any time during the interview if they feel unwilling to participate although with their previous consent. The audio records and transcripts were safely protected till the end of the study and only the researcher has had access to them. After the completion of the study the audio files were deleted. The quotes of participants have been coded without including their personal information.

### 3.5 Data analysis

Data were analysed by using content analysis according to Krippendorff method (2004) to describe work stress and its sources among oncology nurses working in a Saudi university‐teaching hospital. In this method, there are some important steps involving unitizing, reduction, inference, and interpretation of data as well as making conclusion. Unitizing was established after repeated reading of transcripts until the investigator developed a general sense of the data. Then, the words, sentences, and paragraphs which had special meanings and the answers to the study questions were identified (unit of analysis). Also, this step was involved to establish the coding frame. For this purpose, the investigator has marked participants’ perceptions of work stress and it sources, after reading the transcripts for numerous times. The investigator had extracted the representative units of data and placed them within the sampling framework. Therefore, identifying the frequency of the data, regarding the meanings of the words and sentences which were the same, they were placed under one meaning or structure. By the progress of the analysis, the data reduction occurred, and data categories were developed. In advanced stages of inference and conclusion, the investigator interpreted the findings and writing data to text.

### 3.6 Rigour

To establish credibility and trustworthiness, peer debriefing and member checking were conducted (Holloway, 1997; Thomas & Magilvy, 2011). In member checking, the participating oncology nurses reviewed and confirmed data and the extracted codes. Also, for reviewing the analysis's process, the texts of the interviews, codes, and derived categories were evaluated and verified by academic nurses experienced in qualitative research. In addition, the findings were shared with some oncology nurses who did not participate in this study and they confirmed the appropriateness of the findings.

## 4 FINDINGS

The mean age of participants was 32, ranging from 26‐35. All participants had advanced preparation/specialized education in oncology nursing. Participants had a mean 5.41 years of experience in oncology nursing. Table 2 shows sociodemographic characteristics of oncology nurses.

**Table 2:** Socio‐demographic characteristics of participants

Variable	Number of Participants (*N* = 14)
Gender	
Male	4
Female	10
Country of origin	
Philippines	8
India	4
Saudi Arabia	1
Pakistan	1
Nursing education	
Bachelors of Nursing	9
Nursing Diploma	5
Advanced preparation in oncology nursing	
Yes	14
No	0
Oncology unit/clinic	
Male Medical Oncology	2
Male Surgical Oncology	2
Female Medical Oncology	1
Female Surgical Oncology	2
Paediatric Oncology	2
Gynaecology	2
Haematology/Oncology Clinics	3
	M *SD*
Age	32.46 7.77
Years in nurses experience	10.04 7.06
Years in oncology nurses experience	5.41 3.55

The study findings were interpreted in relation to Lazarus and Folkman's (1984) transactional stress theory. Two main categories about extend of work stress and work‐related stressors were identified. The second category included the following subcategories of workload and staff shortage, emotional demands, lack of social support, language barriers, and lack of respect from patients and family members and cultural differences. Illustrative quotes are reported verbatim.

### 4.1 Category 1: Extend of work stress

Most of the participants, irrespective of gender, expressed high levels of stress in the workplace. Participants attribute this stress to several sources. Some participants explained that work as an oncology nurse is rewarding but the job roles in the Saudi hospital were difficult. One participant reported:

“I would say I was happy to be an oncology nurse. Oncology nursing as a profession itself is very rewarding and emotional work, but the work stressors that I face here make me feel much stressed.” (Interview no. 1)

Another female participant stated:

“working here in this hospital are really stressful and difficult. I am completely tired and exhausted… all I want to do when back home after finishing my duty is sleep.” (Interview no. 3)

### 4.2 Category 2: Work‐related stressors

The participants expressed work stress from several sources. The work‐related stressors are arranged according to the most common ones identified by participates.

#### 4.2.1 Workload and staff shortage

Workload and oncology nursing staff shortage were the most common work‐related stressors among participants. Their experiences showed that high workload and staff shortage can cause stressful consequences, such as physical and mental exhaustion. One participant responded:

“It is a great opportunity to work in oncology unit. However, we work under high levels of stress due to work overload. We handle palliative, haematology and medical oncology patients with more paperwork.” (Interview n. 3)

Two participants indicated that the hospital has high expectations on them. This created an extra workload and they felt somewhat physically and emotionally exhausted, as well as, incompetent to carry out their duties. Another participant to show the negative effects of high workload and staff shortage on patient care and relations, said:

“Because of work overload and lack of staffing, there is no enough time to spend with each patient, to meet his/her needs and to listen to their problems.” (Interview n. 10)

#### 4.2.2 Emotional demands

Emotional demands were considered another common work‐related stressor among participants that cause a strong impact. Participants reported that they always feel attached to their patients because they stay at the hospital for long periods of time. Seven participants admitted that patient's death is the most stressful situation in their clinical practice. One of the participants reported:

“...it is very hard to see patients dying after building a great relationship with them…that affects my emotion and personality.” (Interview n. 7)

This typically also involved the situation where the cancer patient had deteriorated unexpectedly, and all rescue attempts were ineffective. Another participant stated that he feels stressed when the patient's condition worsens, and he feels that he can no longer help his patient. At that time, he experiences a deep sense of weakness. (Interview n. 12)

#### 4.2.3 Lack of social support

Social support defined by participants as the degree to which staff notice that their organization is esteemed by workplace sources, such as head nurses and supervisors. Half of the participants agreed that no support was given to them from their supervisors and there are no good systems in the hospital to support them. One participant said:

“Unfortunately, the organization only cares about work and the patient census but not cares about who provides the care and no support at all.” (Interview n. 2) Another participant added: “No support system to defend nurses at all. Once an issue occurs, it is easy to blame a nurse because it is well known that she is defendless.” (Interview n. 5)

#### 4.2.4 Language barriers

Language barriers were frequently cited as work‐related stressors of some participants. As oncology nurses, they need to communicate effectively with their patients and families and provide them emotional support. Six participants expressed great difficulty in communicating with people with cancer in some situations due to language barriers. According to one participant:

“I have difficulty in commutating with patients and their families…they do not know how to speak English. I have to exert a lot of effort in learning their language…Not understanding what the patient was saying, what someone was asking affected my morale and created stress.” (Interview n. 14)

Another participant commented:

“I can say that it is very hard and difficult to handle oncology patients especially we are not Arabic speakers…it is really difficult to explain things to them and share our feelings toward them.” (Interview n. 3)

They experienced feelings of frustration and helplessness and described these experiences as a source of stress.

#### 4.2.5 Lack of respect from patients and family members

Some participants indicated that people with cancer and their relatives are too dependent on nurses and are not willing to participate in patient care activities and take responsibility; other people with cancer are difficult to manage because they are aggressive or abusive. One participant said that:

“Cancer patient and family in Saudi Arabia not believe in nursing and treat nurses as unprofessional people. Patients are treating us like servants or slaves.” (Interview n. 9)

Another female participant stated:

“Sometimes the family/relatives of patient does not fully understand nurse's role. They think that nurses are just for changing patient's diapers etc… They do not fully understand how a nurses handles a patient with cancer.” (Interview n. 13)

#### 4.2.6 Cultural differences

Culture can be defined as the customary beliefs, values, and ideas held by a group of people (Avruch, 1998). Few participants indicated that family members of people with cancer are difficult to manage because they refuse to follow the nurse's advice, instructions, or hospital rules. This was supported by four of the participants. For example:

“… If a patient has a wound, family is insisting to use their cultural or traditional ways of dressing and refuses to adhere to hospital policy of dressing.” (Interview n. 1)

“Some patients insisted using their own herbal medicine that not advised by the doctor.” (Interview n. 4)

Another aspect of Saudi culture observed by some participants is that a high level of participation, influence, and interference by patients’ families which had an impact on direct care and placed oncology nurses in stressful situations. A specific example of how patient relatives interfere and ask to control the information given to the patient, especially if it was bad news. One participant reported:

“What I realize in working with people with cancer that physicians not told the truth about the patient's condition and cancer treatment due to family request and as a nurse I get in the middle between the physician and my patient.” (Interview n. 11)

Another participant added that:

“When the patient does not know about his/her disease as requested by family members, patient keeps asking me…stressful situation.” (Interview n. 3)

## 5 DISCUSSION

The qualitative interviews explored and provided a richer understanding of work‐related stressors among Registered Nurses working in oncology units/clinics at a university‐teaching hospital in Jeddah, SA. As stated in transactional stress theory developed by Lazarus and Folkman (1984), what carries as either subjective or objective stress resides in the relationship between the environment and the individual that leads to a threat appraisal. This interaction between oncology nurses and work environment plays a major role in participants’ experiences of stress. In particular, workload and staff shortage, emotional demands, lack of social support, language barriers, and lack of respect from patients and family members and cultural differences were identified as sources of work stress that support the theory (stressors resulted from interactions between individuals and their environment, workplace). A stressor was viewed as any event, situation, or person that an individual may encounter in the environment that requires change or adaptation. The model provided a framework in which to view oncology nurses’ stressors and provided a structure to facilitate analysis of the study findings. The participants, irrespective of gender, experienced high levels of work stress in this study. This finding is consistent with the previous quantitative study of Wazqar et al. (2017a) that reported oncology nurses working in five Saudi cancer care settings have high to moderate levels of work stress which lead to poor work performance. On the contrary, in a review conducted by Peters et al. (2012), there was no strong evidence that oncology nurses experience higher levels of work stress than nurses in other disciplines. Brajtman et al. (2007) also argue that experienced nurses who have adequate preparation in oncology nurses, positive attitude towards the profession and using effective stress management skills may not experience stress that affect quality of nursing care. Workload was identified as the highest source of work stress and has been linked to staff shortage among oncology nurses. This finding is consistent with the study by Ko and Kiser‐Larson (2016) in the USA of nurses working in oncology outpatient units, the results of which showed “workload” as a result of staff shortage to be one of the highest work‐related stressors for oncology nurses. Toh, Ang, and Devi (2012) also found a positive, bidirectional relationship between staff shortage and work stress, which has led to a rise in the number of oncology nurses leaving the specialty, thereby compounding the problem further. In another study conducted by Bardeh et al. (2016), researchers found that poor relationships with colleagues is the highest source of work stress among Iranian oncology nurses, thus in disagreement with this study's findings. It might have been due to differences in healthcare systems, types of settings (public vs. private) and oncology units. However, researchers claimed that oncology nurses who have assumed higher levels of workload and tasks with limited number of nursing staffs, become vulnerable to negative outcomes including higher levels of depression, anxiety, and burnout (Escot, Artero, Gandubert, Boulenger, & Ritchie, k., 2001; Karanikola, Giannakopoulou, Kalafati, Kaite, & Patiraki, 2016; Tuna & Bayka, 2014). The second important sources creating stress which were mentioned by the participants of the present study have been referred to emotional demands. Emotional demands can be defined as those aspects of the work that require continuous emotional effort because of interactional contact with patients (de Jonge & Dormann, 2003). This study found that oncology nurses had a strong emotional connection with their people with cancer, explained by the unique nature of the disease and long hospitalization periods. The study conducted by Rodrigues and Chaves (2008) identified the most stressful situations for oncology nurses as dealing with death and dying and low efficacy in relieving the patient's suffering, which agrees with this study’s findings. In another study conducted in SA by Saleh, Saleh, and Abduruz (2013), researchers noted that the most stressful subscale for oncology nurses was death and dying and the least stressful subscale was inadequate preparation to help with the emotional needs of patients and their families. The unpredictable or sudden changes in cancer patient status were among the most stressful experiences that the participants were faced with. Peters et al. (2013) and Escot et al. (2001) have indicated that oncology nurses may benefit from death education, communication, and intervention programmes in the areas of increased self‐awareness, change in attitudes, having a positive attitude towards death and dying, and obtaining the knowledge and skills in providing culturally sensitive care for dying patients. However, Bardeh et al. (2016) noted that among the sources of work stress under the investigation, the lowest stress mean (3.15) with standard deviation of 1.36 is associated with emotional demands in oncology nurses. In addition, there was a nonsignificant relationship between stress and emotional demands in a study conducted by Abdulaziz (2016) among Malaysian oncology nurses working in a public hospital, as the dataset highlighted a positive correlation *r *(0.219) and a *p* value of more than 0.05 (0.175).

Social support refers to interrelated social relations and connections that help in the coping and dealing of individuals with stressful situations (Albar Marin & Garcia‐Ramirez, 2005). A finding from the present study shows that oncology nurses viewed the lack of support from the management of the healthcare organization and supervisors as a major work‐related stressor. The finding is consistent with Hamaideh, Mrayyan, Mudallal, Al‐Faouri, and Khasawneh (2008), who found a significant correlation between nurse work stressors and received social support. Nwozichi and Ojewole (2015) and Willard and Luker (2007), also indicated that support from healthcare organization and nursing administration, could act as a buffer to decrease work stress and is health protective for oncology nurses. None of the previous studies paid attention directly to work stress associated with lack of social support among oncology nurses in SA to compare and contrast findings. Oncology nurses work closely with patients’ family members, informing them about the patient's disease process and discussing with them the patient's treatment plan and common side effects. It would appear from this study's findings that interacting with family members is equally as difficult and demanding as working with Saudi people with cancer, due to language barriers and cultural differences. There was a similar finding in a qualitative study conducted among urban and rural community nurses working with palliative care clients in homes in Australia, showing that issues with the patients and families were perceived by nurses to be the highest source of work stress in oncology nursing (Wilkes & Beale, 2001). Wazqar, Kerr, Regan, and Orchard (2017b) also indicated that common challenges facing oncology nurses in SA are related to communication, language barriers, and cultural differences that may lead to stressful work environments and reduce the quality of care provided to people with cancer. The language barriers between internationally educated nurses and family members of patients lead to further stress and tension between both parties (Halligan, 2006) and increasing incidents of language and cultural misunderstanding (Wyk, 2012). Bardeh et al. (2016) found that among the four work‐related stressors examined, the highest mean is associated with stress in relation to colleagues and the lowest mean is related to stress associated with people with cancer and their families, which disagrees with the current study's findings. In another qualitative study conducted by Simonyan (2017), the study also showed that Armenian oncology nurses have a supportive and positive attitude towards people with cancer and their families and are ready to manage their stress and tension. However, nursing administrators/managers should take these work‐related stressors into consideration to reduce levels of work stress and its sources in Saudi cancer care settings. Participants in this study felt that family members of people with cancer did not have an accurate image of nursing as a profession and treated oncology nurses like servants, which put them in stressful situations. Violence, abuse, lack of cooperation, and disrespect from patients and their families were the stressors which the participants had experienced. Almalki, FitzGerald, and Clark (2011) claimed that the Saudi people do not appreciate the role of nurses in providing health care, considering that nurses are no more than the helpers to physicians. Another study conducted by Hatamleh and Sorio (2017), researchers found that poor interest and preference in nursing compared with other high prestige professional disciplines such as medicine and pharmacology which the Saudi participants preferred. The negative image about nursing in Saudi society is found to negatively affect nurses’ levels of stress and practice (Hatamleh & Sorio, 2017). This finding is inconsistent with a recent descriptive cross‐sectional study, exploring the Saudi community’s perception of nursing as a profession, which found a positive perception about nursing among most participants (Saied, Al Beshi, Al Nafaie, & Al Anazi, 2016). Increase in public awareness about the nursing profession through proper media usage may result in better image of nursing among Saudi individuals and families. In conclusion, there are numerous opportunities for nursing research stemming from this initial qualitative study. A replication of this study with a large sample of oncology nurses from different Saudi hospitals in remote provinces as well as in public and private sectors is needed to compare and contrast findings. Such a study may assist in identifying the overall levels of work stress and its related sources in each sector that may differ from this study's findings and address knowledge gaps. Furthermore, the effects of work stress and its sources on oncology nurses’ mental and physical health in Saudi hospitals has not been, and needs to be, studied. Moreover, the study findings may serve as a resource for developing polices, guidelines, and practices to improve work environments for oncology nurses in SA.

### 5.1 Limitations

This qualitative study had some limitations. The first limitation is sample size which represents a small fraction of oncology nurses working in different cancer care settings in SA. Furthermore, the study focused on a particular region (Jeddah) in SA, which could reduce the generalizability of its findings to other regions. Therefore, the results should be interpreted cautiously when applying them to oncology nurses in other Saudi regions. The nature of qualitative research may make it difficult for other researchers to transfer the results using the same research methods.

## 6 CONCLUSION

By drawing on Lazarus and Folkman's (1984) transactional stress theory, researcher was able to explore a broader range of what constitutes work stress among oncology nurses working in SA. The present study findings clarify that oncology nurses have experienced high levels of work stress and present the common work‐related stressors to which nursing administrative/managers need to give more attention. The effective way to reduce the work‐related stressors among oncology nurses in SA depends largely on hospital administration and human resources’ ability to address these sources and deal with them correctly. Nurse educators in the hospitals may find the study results useful when designing strategies to support oncology nurses develop effective stress management skills to deal with stressful situations at work. For example, increased stress reduction education, such as workshops or seminars for oncology nurses and discussing common work‐related stressors and how to deal with stress effectively (coping and stress reduction skills) may be helpful to reduce oncology nurses’ stress levels and improve their stress management skills. Some actions also seem to be necessary to improve the social status of nursing profession in the Saudi society and media. Quantitative studies are recommended to generalize and confirm the findings of this study on all oncology nurses working in Saudi cancer care settings. Participants of both sexes, diverse cultural groups, and various geographical areas would have been more representative of the population of oncology nurses in SA.

## CONFLICT OF INTEREST

No conflict of interest has been declared by the author.

## AUTHOR CONTRIBUTIONS

All authors have agreed on the final version and meet at least one of the following criteria [recommended by the ICMJE (https://www.icmje.org/recommendations/)]:

- substantial contributions to conception and design, acquisition of data, or analysis and interpretation of data;
- drafting the article or revising it critically for important intellectual content.
